# Orthopantomographic Study of Mandibular Morphology Across Different Growth Patterns: A Retrospective Data Analysis

**DOI:** 10.7759/cureus.90774

**Published:** 2025-08-22

**Authors:** Anjali Gupta, Parul Jain, Abhishek Sharma, Atul Jindal, Ushmita Mehta, Mishal Adnan, Mohammed Ismail, Gurjot Singla, Seema Gupta

**Affiliations:** 1 Department of Orthodontics, Guru Nanak Dev Dental College and Research Institute, Sunam, IND; 2 Department of Orthodontics, Kothiwal Dental College and Research Centre, Moradabad, IND

**Keywords:** growth, mandibular, morphology, orthopantomogram, pattern, retrospective

## Abstract

Introduction: Variations in mandibular morphology may significantly influence orthodontic treatment planning, particularly in different growth patterns. This study aimed to evaluate mandibular morphology using orthopantomogram (OPG) analyses to quantify the differences in ramus height, mandibular body length, gonial angle, and condylar morphology across different growth patterns, thereby providing insights for personalized orthodontic interventions.

Materials and methods: A retrospective cohort study was conducted using the records of 120 subjects with skeletal Class I malocclusion, aged 18-35 years. The participants were divided into three groups (n=40 each) based on the Frankfort-mandibular plane angle (FMA): hypodivergent (<20°), normodivergent (20-30°), and hyperdivergent (>30°). The inclusion criteria were good-quality OPGs and cephalograms, subjects with no history of trauma, temporomandibular joint dysfunction, or previous orthodontic treatment. OPGs were manually traced to measure condylar height, ramus height, corpus height, mandibular body length, gonial angle, and condylar morphology (classified as oval, diamond, bird beak, or crooked finger). The intra- and inter-observer reliabilities were confirmed using intraclass correlation coefficients. The data were statistically analyzed.

Results: Significant differences in mandibular morphology were observed across different growth patterns (p<0.05). The hyperdivergent subjects showed reduced condylar and ramus heights, increased gonial angles, and higher corpus heights, whereas the hypodivergent subjects exhibited increased posterior dimensions and reduced gonial angles. Normodivergent subjects had the longest mandibular body length. The condylar morphology varied, with predominant and atypical oval shapes (bird beak and crooked finger) more prevalent in hyperdivergent cases. Post hoc analyses confirmed distinct intergroup differences.

Conclusion: Mandibular morphology varied significantly in different growth patterns and influenced the ramus height, gonial angle, and condylar shape. These findings underscore the importance of tailored orthodontic strategies based on growth pattern-specific mandibular characteristics to enhance treatment precision and efficacy.

## Introduction

The mandible, or lower jaw, is a critical anatomical structure in the craniofacial complex that plays a pivotal role in mastication, speech, and facial aesthetics. Its morphology, encompassing size, shape, and structural characteristics, is influenced by a combination of genetic, environmental, and functional factors [1]. Understanding mandibular morphology in relation to different growth patterns is essential in orthodontics, maxillofacial surgery, and forensic anthropology because it aids in the diagnosis, treatment planning, and prediction of craniofacial development outcomes [2]. Growth patterns, typically classified as vertical, horizontal, or average, significantly affect mandibular development, leading to variations in form and function [2,3]. These patterns are determined by the interplay of skeletal, dental, and soft tissue relationships, which are often assessed using cephalometric analyses, cone-beam computed tomography (CBCT), or other imaging modalities [2].

Mandibular morphology varied distinctly across the growth patterns. In individuals with a horizontal growth pattern, the mandible often exhibits a more robust and anteriorly positioned structure, characterized by a prominent chin and lower mandibular plane angle. Conversely, vertical growth patterns are associated with a more elongated and posteriorly divergent mandible, often linked to a high mandibular plane angle and a retrognathic profile [2,4]. Average growth patterns present a balanced mandibular morphology with moderate angulation and proportional development [2,5]. These variations influence not only facial aesthetics but also functional outcomes such as occlusion and temporomandibular joint (TMJ) dynamics. Understanding these differences is crucial for orthodontists to tailor interventions, such as functional appliances or orthognathic surgery, to optimize skeletal harmony and occlusal stability.

The evaluation of mandibular morphology relies on advanced diagnostic tools to quantify the structural differences. Orthopantomograms (OPGs) provide a comprehensive two-dimensional (2D) view of the mandible, allowing the assessment of parameters such as mandibular angles, ramus height, and body length [5,6]. Cephalometric radiographs complement OPG by offering additional insights into craniofacial relationships, whereas CBCT provides three-dimensional (3D) visualization of the bone architecture [2,4]. Growth patterns are assessed using parameters such as the Frankfort-mandibular plane angle (FMA), gonial angle, and y-axis, which help categorize individuals into distinct growth types [2,4,5]. For instance, individuals with vertical growth patterns may require interventions to control excessive vertical development, whereas those with horizontal growth patterns may benefit from strategies to enhance their mandibular projection.

The study of mandibular morphology under different growth patterns has implications beyond orthodontics. In forensic anthropology, mandibular characteristics aid in age and sex determination, whereas in evolutionary biology, they provide insights into dietary adaptations and craniofacial evolution [7]. Moreover, understanding mandibular growth is vital for managing congenital anomalies, such as mandibular hypoplasia or asymmetry, which may manifest differently across growth patterns [8]. As research progresses, interdisciplinary approaches combining genetics, biomechanics, and imaging will further elucidate the complex interplay between mandibular morphology and growth patterns, thus paving the way for personalized treatment strategies.

This study aimed to evaluate the mandibular morphology in relation to different growth patterns using OPG and cephalometric analyses. The objective was to quantify variations in mandibular parameters, such as ramus height, body length, gonial angle, corpus height, condylar height, and morphology in vertical, horizontal, and average growth patterns. The null hypothesis stated for the study was that there would not be any significant difference in mandibular morphology across vertical growth patterns and condylar shape distribution would not be associated with specific vertical growth types.

## Materials and methods

This retrospective cohort study was conducted at the Department of Orthodontics of Guru Nanak Dev Dental College and Research Institute in Sunam, Punjab, India, from May 2023 to December 2024. Ethical approval was obtained from the Institutional Ethics Committee of the college (approval number: GNDDC/IEC/2023/012), ensuring compliance with ethical guidelines. Written informed consent was obtained from all subjects prior to their radiographic records.

A priori power analysis was conducted using G*Power Version 3.1.9.2 (Heinrich-Heine-Universität Düsseldorf, Düsseldorf, Germany) using one-way analysis of variance (ANOVA) with an effect size of 0.28 (derived from a prior study analyzing gonial angle differences among growth patterns) [3]. The estimated sample size required to achieve 80% power at a 5% alpha error level was 120 (40 per group).

The records of 400 subjects seeking orthodontic treatment at the department were screened, of which 120 were selected based on the specific eligibility criteria. The inclusion criteria were subjects with skeletal Class I malocclusion (ANB angle 0-4°), presence of good-quality OPGs and lateral cephalograms, age 18-35 years, no history of trauma, TMJ dysfunction, recent occlusal adjustments, or previous orthodontic treatment. Subjects with unilateral or bilateral crossbite, syndromic features, subdivision malocclusion, or mandibular deviation during closure were excluded from the study.

The selected samples were divided into three equal groups (n=40) based on different growth patterns determined by the FMA: Group A (hypodivergent, FMA <20°), Group B (normodivergent, FMA 20-30°), and Group C (hyperdivergent, FMA >30°). Radiographic imaging was performed using Carestream CS 8000C (Carestream Health, Rochester, New York, United States) for both OPGs and lateral cephalograms using Carestream radiographic films (20×25 cm). The films were exposed at 74 kVp and 12 mA for 0.8 seconds and developed using a laser imager (DryView 5700, Carestream Health, Rochester, New York, United States) by a single trained technician to ensure consistency. Lateral cephalograms were obtained with a cephalostat, positioning the patients in a natural head position with the teeth in centric occlusion. OPGs were obtained by following standard protocols for capturing the entire mandibular structure. All radiographs were manually traced by a single investigator on a 36 micrometer matte acetate tracing paper (OrthoTrace, Ortho Organizers, Carlsbad, California, United States) secured with adhesive tape (3M, St. Paul, Minnesota, United States) on a light box (Huion L4S, Shenzhen, China). Tracings were performed using a 3H microlead pencil (Staedtler, Nuremberg, Germany) with geometric tools, including a set square, protractor, compass, and metallic scale (Faber-Castell, Stein, Germany) for precise measurements.

**Figure 1:**
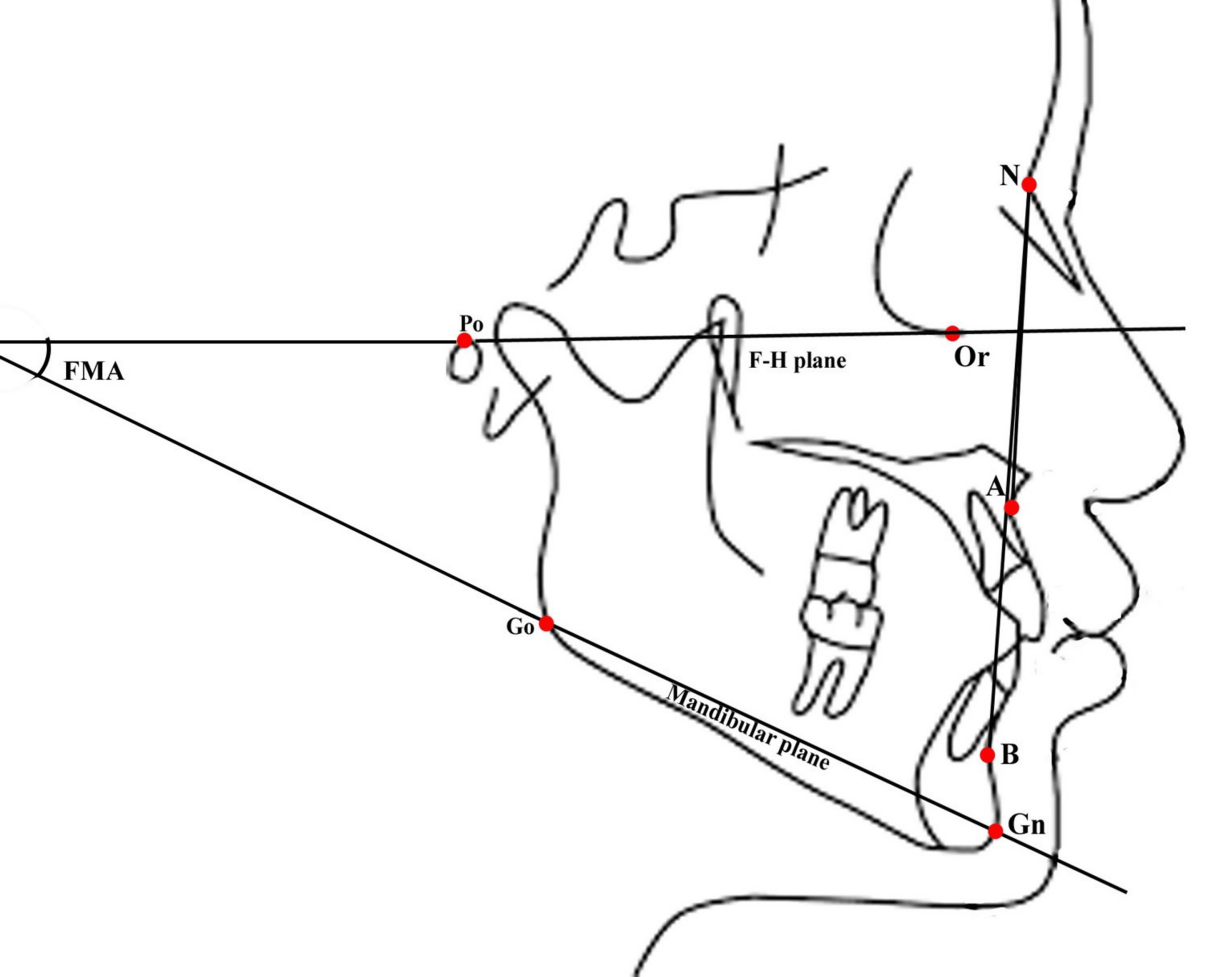
Landmarks identified on a lateral cephalogram The ANB angle, formed by points N, A, and B, is used to assess skeletal relationships. The FMA is formed between the F-H plane (passing through the Po and Or points) and the mandibular plane (passing through the Go and Gn points) to evaluate growth patterns. N: Nasion; FMA: Frankfort-mandibular plane angle; F-H plane: Frankfort-horizontal plane; Po: Porion; Or: Orbitale; Go: Gonion; Gn: Gnathion This figure is an original image created by Anjali Gupta.

**Figure 2:**
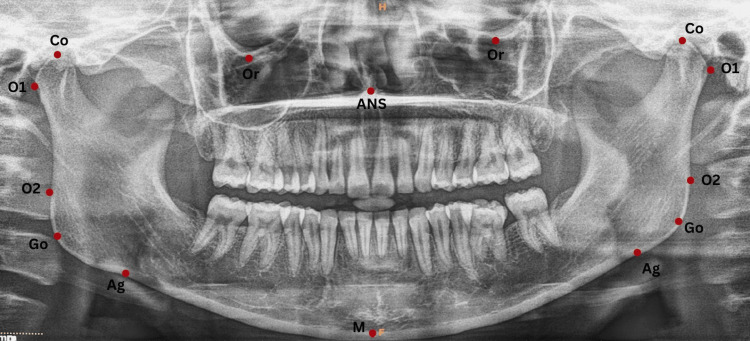
Landmarks identified on an orthopantomogram Or: Orbitale; Co: Condylion; Go: Gonion; Ag: Antegonion; M: midpoint on the inferior border of the mandible; ANS: anterior nasal spine; O1: the most lateral point on the condyle; O2: the most lateral point on the ascending ramus This figure represents the original orthopantomogram of the patient, obtained with the patient's permission, from the study.

Landmarks and planes were identified on lateral cephalograms (Figure 1) and OPGs (Figure 2). The details of the landmarks and planes are provided in Table 1.

**Table 1:** Landmarks and planes used in the study Go: Gonion; Gn: Gnathion; Po: Porion; Or: Orbitale; Co: Condylion; F-H plane: Frankfort-horizontal plane; Ag: Antegonion; M: mandibular midpoint; ANS: anterior nasal spine

S. no.	Landmarks and planes	Description
1	Point A	The deepest point on the concavity of the anterior maxillae
2	Point B	The deepest point on the concavity of the anterior mandible
3	Point N	The most anterior point on the frontonasal suture
4	Go	The most posteroinferior point located at the angle of the mandible
5	Gn	The most anteroinferior point on the bony chin
6	Po	The most superior point on the external auditory meatus
7	Or	The lowermost point on the bony orbit
8	Co	The uppermost point of the condyle
9	F-H plane	A plane passing through points Po and Or
10	Mandibular plane	A plane passing through points Go and Gn
11	O1	The most lateral point on the condyle
12	O2	The most lateral point on the ascending ramus
13	Line A	A line connecting O1 and O2
14	Line B	The perpendicular line from Line A to the superior part of the condyle
15	Ag	The uppermost point of the concave notch along the lower border of the mandibular ramus where it merges with the body of the mandible
16	M	Identified by projecting the mental spine into the inferior border of the mandible, at its intersection with the vertical plane passing through the ANS
17	ANS vertical plane	A vertical line drawn from the ANS perpendicular to the Or plane (a plane passing through right and left Or points)

Angular measurements included the ANB angle (to confirm skeletal Class I malocclusion) and the FMA (to classify growth patterns) on lateral cephalograms (Figure 1 and Table 2).

**Table 2:** Angular parameters used in the study Co: Condylion; Go: Gonion; M: midpoint on the inferior border of the mandible; N: Nasion; FMA: Frankfort-mandibular plane angle; F-H plane: Frankfort-horizontal plane

S. no.	Parameters	Description
1	Gonial angle (°)	An angle formed by two lines: one tangent to the lower border of the mandible and the other tangent to the posterior border of the ramus
2	Co-Go-M angle (°)	An angle formed by connecting points Co, Go, and M points, measured at Go
3	ANB angle (°)	An angle formed between A, N, and B points to measure skeletal pattern
4	FMA (°)	An angle formed between F-H plane and mandibular plane to assess growth pattern

**Figure 3:**
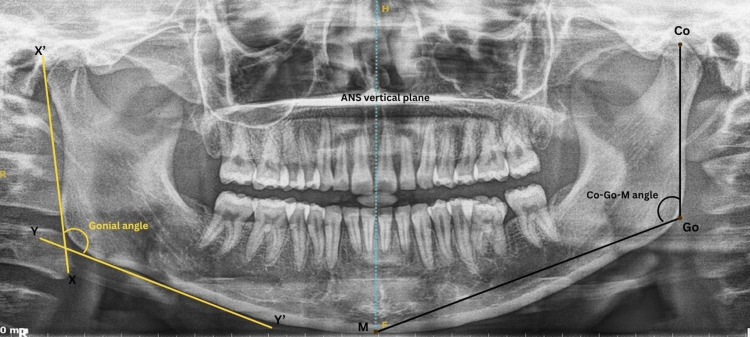
Angular parameters traced on an orthopantomogram The gonial angle is formed between the ramal plane (XX') and the mandibular plane (YY'). The Co-Go-M angle is formed by the points Co, Go, and M. Co: Condylion; Go: Gonion; M: midpoint on the inferior border of the mandible at its intersection with the anterior nasal spine vertical plane This figure represents the original orthopantomogram of the patient, obtained with the patient's permission, from the study.

The gonial angle and Co-Go-M angle were traced on OPG (Figure 3 and Table 2).

**Figure 4:**
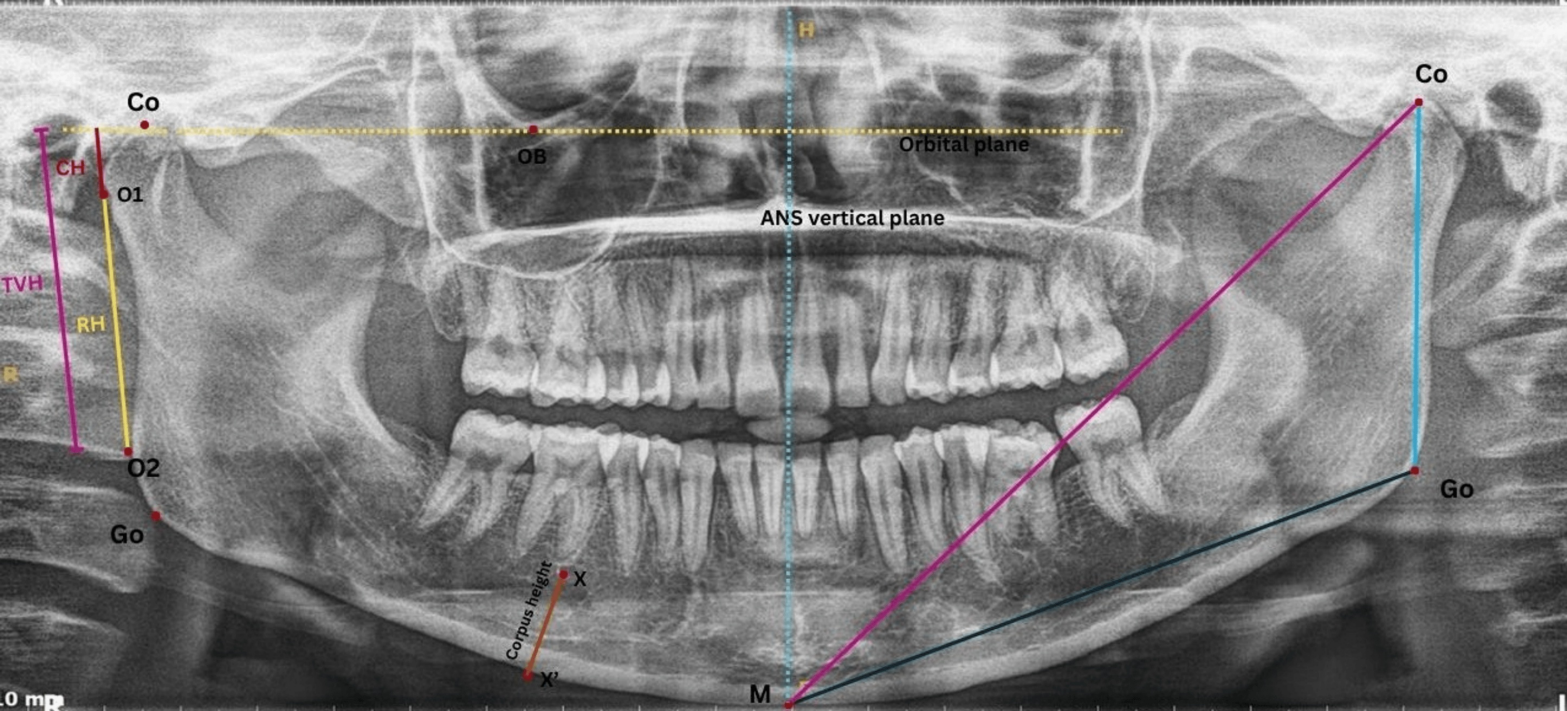
Linear parameters traced on an orthopantomogram CH represents condylar height (mm), measured as the red line from Line B through the Co point to O1. RH represents ramal height (mm), measured as the yellow line from O1 to O2 (Line A). TVH represents total vertical height (mm), measured as the purple line from Co to O2. Corpus height (mm) is measured as the red line from X (apex of the mesial root of the first molar) to X' (lower border of the mandible). Co-M represents total mandibular length (mm), measured as the purple line from Co to M. Go-M represents mandibular body length (mm), measured as the black line from Go to M. Co-Go represents combined condylar and ramal height (mm), measured as the blue line from Co to Go. Co: Condylion; M: midpoint on the inferior border of the mandible; Go: Gonion This figure represents the original orthopantomogram of the patient, obtained with the patient's permission, from the study.

For OPGs, different linear parameters were evaluated, including condylar height, ramus height, total vertical height, mandibular length, mandibular body length, and height of the corpus (Figure 4 and Table 3).

**Table 3:** Linear parameters used in the study to assess mandibular morphology on orthopantomogram CH: condylar height; RH: ramus height; TVH: total vertical height; Go: Gonion; M: midpoint on the inferior border of the mandible; Co: Condylion

S. no.	Parameters	Description
1	CH in mm	The vertical distance from the Line A to O1 point was measured
2	RH in mm	The distance between O1 and O2 was measured
3	TVH in mm	The distance from Line A to the O2 point was measured
4	Length of the mandibular body (Go-M) in mm	The distance measured from point Go to point M
5	Height of the corpus in mm	The distance between the apex of the mesial root of the mandibular first molar and the inferior border of the mandible
6	Total mandibular length in mm (Co-M)	The distance measured from point Co to point M
7	Co-Go in mm	The distance measured from point Co to point Go as condylar plus ramal height

Condylar morphology was classified as oval, diamond, bird beak, or crooked finger shape [9]. All the measurements were performed bilaterally, and average values were taken for the analyses. To ensure reliability, a calibration process was conducted prior to data collection. Two investigators, including the primary investigator, were trained to identify landmarks and consistently perform measurements. Intra-observer reliability was assessed by retracing 15 randomly selected OPGs and cephalograms after a two-week interval, with measurements compared using the intraclass correlation coefficient (ICC). An ICC value above 0.9 was achieved for all landmarks and measurements. Inter-observer reliability was evaluated by having a second investigator independently trace and measure 10 randomly selected radiographs, with results compared to the primary investigator's using paired t-tests, showing no significant differences (p>0.05).

Data were entered into Microsoft Excel (Microsoft Corporation, Redmond, Washington, United States) and analyzed using IBM SPSS Statistics for Windows, Version 26.0 (Released 2019; IBM Corp., Armonk, New York, United States). Normality was confirmed using the Shapiro-Wilk test, with a p-value below 0.05, permitting parametric tests. Continuous data were presented as means and standard deviations, while categorical data were presented as frequencies and percentages. Continuous variables were compared across the three groups using one-way analysis of variance (ANOVA) with Tukey's post hoc test for pairwise comparisons. Categorical variables were analyzed using Fisher's exact test. Statistical significance was defined as a two-tailed p-value of <0.05. All measurements were performed by the primary investigator to minimize variability.

## Results

The study included 120 subjects (40 per group) with mean ages of 28.2±5.9 years for hypodivergent, 27.5±7.2 years for normodivergent, and 24.4±4.3 years for hyperdivergent. The sex distribution varied across groups, with males predominating in hypodivergent cases and females in hyperdivergent cases. As expected, the FMA differed significantly among groups, validating the growth pattern classification (Table 4).

**Table 4:** Baseline demographic characteristics of the sample Age and FMA are presented as mean and standard deviation (SD), and sex is presented as frequency (n) and percentage (%), where n denotes the number of subjects. FMA: Frankfort-mandibular plane angle

Parameters		Hypodivergent	Normodivergent	Hyperdivergent
Male	n (%)	24 (60)	20 (50)	16 (40)
Female	n (%)	16 (40)	20 (50)	24 (60)
Age (years)	Mean±SD	28.2±5.9	27.5±7.2	24.4±4.3
FMA (degrees)	Mean±SD	17.5±3.45	26.5±5.35	35.52±4.65

The null hypotheses were rejected for the study. The study revealed significant differences in mandibular morphology across growth patterns (p<0.05). Hyperdivergent subjects exhibited significantly reduced condylar, ramus, and total vertical heights, suggesting a diminished vertical development. The gonial angle was markedly increased in the hyperdivergent cases, consistent with the skeletal pattern. Notably, the corpus height increased progressively from hypo- to hyperdivergent, possibly compensating for the reduced ramus dimensions. In contrast, hypodivergent subjects showed increased condylar height, ramus height, and total vertical height, reduced gonial angle, and increased mandibular body length. Normodivergent subjects had the longest mandibular body length and length of the coronoid process. The rest of the measurements were more than those of the hypodivergent subjects, but less than those of the hyperdivergent subjects. Post hoc analysis further confirmed distinct intergroup differences, while the condylar shape showed marginal significance (p=0.034). Non-significant parameters, such as Co-Go length (p=0.067), implied minimal growth pattern influence on certain dimensions. These findings underscore the profound impact of vertical growth patterns on mandibular architecture, with hyperdivergence associated with shorter posterior heights and adaptive anterior development, which are critical for orthodontic and surgical planning (Table 5).

**Table 5:** Comparison of mandibular morphological parameters between different growth patterns using one-way analysis of variance followed by post hoc analysis by Tukey's test Data are presented as mean and standard deviation (SD). *p<0.05 denotes statistical significance; Hy denotes the hypodivergent group; Nor denotes the normodivergent group; Hp denotes the hyperdivergent group. Co: Condylion; Go: Gonion; M: midpoint on the inferior border of the mandible

Mandibular parameters	Hypodivergent	Normodivergent	Hyperdivergent	F value	P-value	Post hoc analysis		
	Mean±SD	Mean±SD	Mean±SD			Hy vs. Nor	Hy vs. Hp	Nor vs. Hp
Condyle height (mm)	7.69±0.96	7.20±1.23	6.46±1.04	13.08	0.001	0.11	0.001	0.007
Ramus height (mm)	42.36±2.34	42.35±3.45	38.98±4.30	12.7	0.001	0.99	0.001	0.001
Total vertical height (mm)	50.05±2.59	49.55±3.42	45.44±4.28	20.91	0.001	0.79	0.001	0.001
Gonial angle (degrees)	116.09±2.94	120.15±2.88	127.25±3.04	146.2	0.001	0.001	0.0001	0.001
Co-Go-M angle (degrees)	110.34±3.13	115.45±5.41	118.22±4.22	33.7	0.001	0.001	0.001	0.014
Height of the corpus (mm)	12.26±1.22	13.81±1.89	15.81±2.40	35.12	0.001	0.001	0.001	0.001
Co-Go (mm)	58.81±4.44	58.24±4.50	56.36±3.36	2.75	0.067	0.99	0.108	0.108
Co-M (mm)	127.32±8.89	132.04±8.02	125.50±4.29	8.45	0.004	0.013	0.518	0.003
Go-M (mm)	97.02±8.72	103.5±8.29	89.5±4.78	35.19	0.001	0.007	0.001	0.001

Analysis of condylar morphology revealed significant variations among the different vertical growth patterns (p=0.002). Oval shape was predominant across all groups, indicating that it was the most common condylar form. Notably, the bird beak configuration appeared exclusively in the hypo- and hyperdivergent groups, whereas the diamond shape was observed only in the hypo- and normodivergent subjects. The crooked finger variant was uniquely present in hyperdivergent cases. These findings suggest that while oval morphology represents the normative condylar shape, certain atypical configurations (bird beak, diamond) may be associated with specific growth patterns. The exclusive appearance of crooked finger morphology in hyperdivergent individuals may reflect adaptive changes in the condylar growth associated with increased vertical dimensions. These morphological variations could have implications for TMJ function and stability, particularly in hyperdivergent cases where both bird beak and crooked finger morphologies were observed (Table 6).

**Table 6:** Comparison of mandibular condylar shape between different growth patterns with Fisher's exact test Data are presented as n (%), where n denotes the number of subjects. *p<0.05 denotes statistical significance.

Shape of the condyle	Frequency (percentage)	Hypodivergent	Normodivergent	Hyperdivergent	Test statistics	P-value
Bird beak	n (%)	4 (10)	0 (0)	4 (10)	20.34	0.002*
Crooked finger	n (%)	0 (0)	0 (0)	4 (10)		
Diamond	n (%)	8 (20)	8 (20)	0 (0)		
Oval	n (%)	28 (70)	32 (80)	32 (80)		

## Discussion

This study provided a detailed evaluation of mandibular morphology across hypodivergent, normodivergent, and hyperdivergent growth patterns using OPGs. The findings revealed significant variations in mandibular parameters such as ramus height, mandibular body length, gonial angle, and condylar morphology, which are closely associated with vertical growth patterns. This study demonstrated that mandibular morphology varied significantly across growth patterns. Hyperdivergent subjects exhibited reduced condylar height, ramus height, and total vertical height, along with an increased gonial angle, reflecting a characteristic posterior mandibular rotation and a steeper mandibular plane. These findings align with those of previous studies, which suggested that hyperdivergent growth is driven by limited vertical development of the condylar cartilage, leading to a more obtuse gonial angle [2-4]. For instance, Buschang et al. [10] noted that hyperdivergent individuals often display a skeletal open bite tendency owing to posterior growth deficiencies, which is consistent with the reduced ramus and condylar heights observed here.

In contrast, hypodivergent subjects showed increased condylar height, ramus height, total vertical height, and mandibular body length with a reduced gonial angle, indicating anterior mandibular rotation and robust posterior development. This was supported by Björk and Skieller [11], who described anterior rotation in hypodivergent patterns as a result of enhanced condylar growth and forward mandibular positioning. The increased mandibular body length in this group may have served to maintain the facial balance in a flatter mandibular plane. The reduced gonial angle observed in our study was supported by previous studies [2,4,12].

Normodivergent subjects displayed the longest mandibular body length and coronoid process, reflecting a balanced growth pattern that optimized both vertical and horizontal dimensions. This finding corroborates previous studies that highlighted intermediate mandibular characteristics in normodivergent individuals, bridging the extremes of hypo- and hyperdivergent patterns [2,4]. The progressive increase in corpus height from the hypodivergent to the hyperdivergent groups is particularly noteworthy. This may represent an adaptive response to reduced ramus height in hyperdivergent individuals, ensuring structural support for masticatory function, as proposed by Frost's mechanostat theory [13], which suggests that bone remodeling adapts to functional demands.

Condylar morphology also varied significantly, with the oval shape predominant across all groups, consistent with previous studies [6,14], which identified the oval as the most common condylar form. The exclusive presence of bird beak morphology in hypo- and hyperdivergent groups, diamond shapes in hypo- and normodivergent groups, and crooked finger morphology in hyperdivergent subjects suggests growth-specific biomechanical influences on condylar development. The crooked finger variant in hyperdivergent cases may reflect altered TMJ loading due to increased vertical dimensions, as noted by Kajii et al. [15], who reported osseous changes in the condyle in skeletal Class II patients with backward rotation of the mandible, leading to an increased gonial angle, potentially impacting joint stability. These variations underscore the interplay between growth patterns and mandibular morphology, which influences both the diagnostic and therapeutic approaches. Similarly, Kjellberg [16] reported a small retrognathic mandible with a steep mandibular plane angle in children with juvenile rheumatoid arthritis.

These findings likely stem from the differential growth dynamics in the condylar cartilage and mandibular symphysis. In hyperdivergent individuals, reduced condylar growth may result from genetic predispositions or environmental factors, such as airway obstruction, leading to posterior rotation and a larger gonial angle, as supported by Lessa et al. [17]. Conversely, hypodivergent growth may be driven by enhanced condylar activity, promoting anterior rotation and increased posterior height. The balanced morphology of the normodivergent group likely reflects the optimal growth conditions, minimizing extreme skeletal deviation. These patterns are further influenced by functional factors, such as muscle activity and occlusal forces, which shape mandibular remodeling, as described by Moss and Salentijn [18] in their functional matrix hypothesis.

Clinical implications

Variations in mandibular morphology across growth patterns have direct implications in orthodontic treatment planning. Hyperdivergent patients with reduced posterior heights and increased gonial angles may benefit from vertical control strategies, such as high-pull headgear or temporary anchorage devices, to manage open bite tendencies, whereas hypodivergent patients may require space management due to increased mandibular body length. Condylar morphology variations, particularly atypical forms in hyperdivergent cases, require TMJ assessment to ensure joint stability during treatment. These findings highlight the importance of tailored interventions based on growth pattern-specific mandibular characteristics to optimize the treatment outcomes.

Limitations

The retrospective design and focus on skeletal Class I malocclusion limit the ability of the study to establish causality and generalizability to other malocclusion types. The modest sample size, reliance on 2D imaging, and potential confounding factors such as genetics or functional habits may have affected the robustness of the findings. Future studies should incorporate 3D imaging, larger and more diverse samples, and longitudinal designs to enhance our understanding of the mandibular morphology across different growth and skeletal patterns.

## Conclusions

This study elucidated distinct variations in mandibular morphology across hypodivergent, normodivergent, and hyperdivergent growth patterns, assessed using OPGs and lateral cephalometric analyses. Hyperdivergent subjects exhibited reduced condylar height, ramus height, and total vertical height along with an increased gonial angle, whereas hypodivergent individuals displayed increased posterior dimensions and a reduced gonial angle. Normodivergent subjects demonstrated balanced mandibular proportions, with the longest mandibular body length and the coronoid process. The condylar morphology varied significantly, with oval shapes predominating and atypical forms, such as bird beaks and crooked fingers, linked to specific growth patterns. These findings highlight the influence of vertical growth patterns on mandibular architecture and provide valuable insights for tailoring orthodontic and orthognathic treatment strategies according to individual skeletal characteristics. The observed morphological differences underscored the importance of integrating cephalometric and OPG analyses into diagnostic protocols to enhance treatment precision and efficacy.
